# Moral Limits of Brain Organoid Research

**DOI:** 10.1177/1073110519897789

**Published:** 2020-01-19

**Authors:** Julian J. Koplin, Julian Savulescu

**Affiliations:** **Julian J. Koplin, Ph.D.**, is a Research Fellow with the Biomedical Ethics Research Group, Murdoch Children's Research Institute and Melbourne Law School, the University of Melbourne. He has a broad range of interests across the field of philosophical bioethics. **Julian Savulescu, M.B., B.S., Ph.D., M.A.**, is Uehiro Chair in Practical Ethics, University of Oxford. He directs the Oxford Uehiro Centre for Practical Ethics and co-directs the Wellcome Centre for Ethics and Humanities. He is Visiting Professorial Fellow in Biomedical Ethics at the Murdoch Children's Research Institute, Melbourne and Distinguished International Visiting Professor in Law at the University of Melbourne.

## Abstract

Brain organoid research raises ethical challenges not seen in other forms of stem cell research. Given that brain organoids partially recapitulate the development of the human brain, it is plausible that brain organoids could one day attain consciousness and perhaps even higher cognitive abilities. Brain organoid research therefore raises difficult questions about these organoids' moral status – questions that currently fall outside the scope of existing regulations and guidelines. This paper shows how these gaps can be addressed. We outline a moral framework for brain organoid research that can address the relevant ethical concerns without unduly impeding this important area of research.

The organoid field is booming. Labelled Method of the Year 2017 by *Nature Methods*,^1^ organoids are self-organising 3D structures, generated *in vitro* from stem cells, that resemble *in vivo* organs in terms of their structure and function. Organoid technology can be used to create *in vitro* models of many parts of the body, including the human brain.

Like other forms of organoid research, brain organ-oid research promises to yield important insights into human biology and disease. Brain organoids can be used to study the early development of the human brain, improve our understanding of neurodevelop-mental disorders, test the pharmacologic effects and toxicity of drugs that interact with neural tissue, and potentially even develop personalized treatments for patient-specific neurodevelopmental disorders. Brain organoid research has enormous potential to contribute to human well-being. Indeed, the field has already seen major achievements, including the use of brain organoids to model neurodevelopmental alterations associated with Zika virus-induced microcephaly,^2^ idiopathic autism,^3^ and schizophrenia.^4^ We therefore have a moral imperative to pursue brain organoid research.

Brain organoid research nonetheless raises important ethical questions. Like other forms of organoid research, brain organoid research raises questions around the type of consent that should be required from tissue donors, the extent to which organoid models should displace research with embryos, fetuses, or nonhuman animals, and how the use of organoids in precision medicine should be regulated. The bio-ethical work needed to resolve these issues is already underway.^5^ However, brain organoid research also raises novel ethical questions not seen in other areas of organoid research — questions that are related to the consciousness and moral status of these entities. Given that some brain organoids partially recapitulate the development of the human brain, it is plausible that “mature” whole brain organoids could one day attain sentience, and perhaps even higher cognitive abilities. Should we should place any restrictions on this area of research, given this potential?

Recent scientific developments have brought these ethical questions to the fore. In late 2018, Researchers from the University of California, San Diego, published the creation of brain organoids that spontaneously produce brain waves resembling those found in premature infants,^6^ prompting widespread discussion about the moral limits of brain organoid research.^7^ Although the electrical activity seen in these organoids does not mean they are conscious, these developments do suggest a growing need to think about the possibility that brain organoids might one day acquire consciousness.

Ethical analysis of brain organoid research is still in its early stages. Many commentators have argued that brain organoids could attain some degree of moral status if they develop characteristics such as consciousness, active pain pathways, or self-awareness. These concerns have led many to suggest that we ought to introduce new limits to brain organoid research (beyond those that apply to stem cell research in general) in order to prevent unethical forms of experimentation.^8^ However, while it is increasingly recognized that research with conscious brain organoids should face *some* restrictions, little progress has been made on identifying what form these restrictions should take.

Nor have existing regulatory frameworks caught up with this emerging set of ethical issues. In Australia, organoid research is regulated by the National Health and Medical Research Council's National Statement on Ethical Conduct in Human Research (while additional restrictions apply if the research uses embryonic stem cells). The National Statement outlines requirements for the collection and use of human biospecimens for research, informed consent requirements from tissue donors, and the communication of research findings or results to participants.^9^ In other words, the Statement ensures that researchers address ethical issues connected to the well-being of tissue donors. It does not consider the question, unique to brain organoid research, of whether we need to consider the well-being of the organoids themselves.

Given that some brain organoids partially recapitulate the development of the human brain, it is plausible that “mature” whole brain organoids could one day attain sentience, and perhaps even higher cognitive abilities. Should we should place any restrictions on this area of research, given this potential?

US regulation of brain organoid research likewise fails to address the unique ethical questions associated with the consciousness and well-being of brain organoids. As in Australia, the relevant regulations and guidelines address issues related to the provenance, procurement, and handling of human tissue. There are no specific research limits based on the moral status of the brain organoids themselves.^10^ Nor do the guidelines of the International Society for Stem Cell Research (ISSCR) currently address these emerging ethical issues. Interestingly, the current ISSCR guidelines *do* recommend specialized ethics committee oversight of both human embryo research and research involving “embryo-like structures that might manifest human organismal potential.”^11^ However, brain organoids lack this potential and would therefore fall within the gaps of this definition even if they had the potential to develop consciousness.

For now, these ethical issues remain in the future. Although brain organoids are rapidly increasing in complexity, there are important structural and functional differences between normal adult human brains and the kinds of brain organoids that have been created to date. Some of these differences are relevant to the prospect of brain organoids achieving consciousness. For example, although brain organoids exhibit neural connections and electrical activity, they have so far failed to form even basic synaptic circuits — without which consciousness is probably impossible.^12^ At the same time, researchers are still developing techniques to achieve the vascularization of brain organ-oids, without which they will not be able to develop beyond their current very small size.^13^ Brain organoid development to the point of consciousness remains a future prospect.

For this reason, the use of *current* brain organoid models does not raise ethical concerns beyond those associated with our treatment of human biospecimens more generally. Indeed, given the potential benefits of this research, there are moral imperatives not to unnecessarily obstruct it. Yet it is not far-fetched to think that brain organoids could one day acquire consciousness — and perhaps even develop more sophisticated cognitive capabilities — as the field continues to develop. We ought to determine the moral limits of organoid research before we pass this point.

## Consciousness in Brain Organoids

We can begin by distinguishing between the kinds of brain organoids that raise concerns about moral status from those that do not. A clear moral line can be drawn at the onset of (phenomenal) consciousness — i.e., the point at which an entity can have experiences, or at which there is *something it is like* to be that entity.^14^ This is the first point at which brain organoids might become capable of suffering, and the first point at which they might develop some degree of moral status.

It is worth noting that because the brain itself does not have pain receptors, brain organoids might not be vulnerable to pain. However, it may be that meninges — which do have pain receptors — also develop. Moreover, we are discussing suffering generally, which is not limited to the experience of pain. For example, brain organoids might suffer from sensory deprivation. Given this potential for suffering, the onset of consciousness is the first point at which brain organ-oids are due moral consideration in their own right. Here, then, is an important moral threshold: we can treat brain organoids according to existing regulatory frameworks for stem cell research *until the point at which organoids develop consciousness*, but we should restrict the kinds of research that can take place beyond this point.

To enforce restrictions based on consciousness, we will need some means of deciding whether brain organoids are (or could plausibly be) conscious. It is not immediately obvious how consciousness could be detected in an *in vitro* model of the human brain, given that such an entity can neither communicate with us directly nor exhibit the kinds of behaviours that suggest conscious experience. How, then, should we determine whether a brain organoid might be conscious, and might therefore possess some degree of moral status?

One option is to look at *structure.* We could extrapolate from the threshold at which human fetuses begin to develop consciousness. We might have reasonable concerns about the consciousness of brain organoids that have reached an equivalent point of morphological development. Some estimates place the onset of consciousness at around 25 weeks' gestational age,^15^ and the beginning of pain perception at around 30 weeks,^16^ but there is arguably sufficient uncertainty about the exact threshold that we cannot rule out the possibility that sentience begins as early as 20 weeks' gestational age.^17^ Assuming this is correct, we can be reasonably confident that a brain organoid lacks even a rudimentary form of consciousness until it resembles the brain of a fetus at 20 weeks' development. Beyond this point, we should treat brain organoids as if they could plausibly possess some degree of consciousness.

An alternative functional approach could involve measuring physical processes that suggest consciousness. For example, we could measure the brain's electroencephalographic responses to magnetic stimulation; the complexity of the brain's response might track the organoid's degree of consciousness.^18^ In principle, such metrics could provide a more fine-grained basis for research limits than extrapolating from structure. We endorse the development of such metrics. In the meantime, the extent to which brain organoids resemble the brains of human fetuses at the onset of consciousness could provide a useful, albeit coarse-grained, approach to determining whether consciousness is a realistic possibility.

Many philosophers argue — we think convincingly — that if we are unsure whether a particular being is conscious we should not treat them as if they lack moral status, but instead err on the side of generosity and treat them as if they have at least partial moral status.^19^ We should not treat brain organoids as mere biological material if they could plausibly be conscious, even if we are not *certain* whether they possess consciousness. Beyond this point, experimentation with brain organoids is not merely experimentation with human tissue. It is experimentation with an entity that may have interests that matter for its own sake — i.e., an entity with moral status.

## Research Limits Based on Consciousness

Would it be ethically permissible to experiment with brain organoids that are (or could potentially be) conscious — for example, to study disorders of the adult or ageing brain? Some people believe, on deontological grounds, that morality includes absolute prohibitions on harming or killing persons without their consent. Should we reject any experimentation with conscious brain organoids on these grounds? No. These absolute moral constraints are usually thought to apply only to *persons.* Personhood is generally thought to require one to have complex cognitive capacities such as autonomy, rationality, emotionality, moral agency, and/or sophisticated self-awareness.^20^ Conscious brain organoids are unlikely to develop such capacities, especially in the absence of meaningful interactions with the outside world. Brain organoids would also lack other features that are sometimes taken to undergird personhood, such as membership in our human community or participation in the social contract.^21^ Unlike human fetuses, human brain organoids would presumably lack even the potential to become persons at a later stage of development. In principle, some forms of experimentation with brain organoids might therefore be morally legitimate *even if* these organoids are conscious.

Indeed, many existing practices presuppose that consciousness *per se* confers neither an absolute right to life nor an absolute right against harmful treatment. Consider, for example, our treatment of nonhuman animals. Mice, rats, and other research animals possess consciousness, but we nonetheless allow many forms of animal research that harm these (and other) animals in order to promote human ends. Although many people — and arguably most philosophers writing on the subject — argue that research animals deserve greater protections, most also hold that it is at least *sometimes* justifiable to harm research animals in order to promote human ends.^22^ We can also draw a parallel with late term abortion. As we have seen, consciousness in human fetuses is generally thought to emerge between 20 and 30 weeks' gestational development. However, most jurisdictions permit abortions beyond this threshold. Even in the case of human fetuses, then, consciousness is not generally thought to confer an absolute right to life. Personhood — not consciousness — is the relevant moral yardstick.

We should nonetheless recognize some moral limits to research with conscious brain organoids. Brain organoids are not persons, but if they are conscious they may nonetheless have interests that could be set back by experimentation — for example, interests in achieving experiential well-being and avoiding suffering. These interests ought to be taken into account. Some principles for doing so can be found in the Three R's — Reduce, Refine, Replace — approach to animal welfare,^23^ which is already well entrenched in animal research guidelines. These three principles can be adapted as follows. First, we should reduce the number of conscious brain organoids used in research to the minimum number necessary to achieve the study's scientific aims. Second, we should refine experimental techniques to reduce possible harms — for example, by administering anaesthetic before experimentation in cases where there is some prospect for pain. Third, in line with the principle of replacement, researchers should use brains organoids only if the aims of the research cannot be realized using non-conscious organoids or other non-sentient material.

Despite its important historical legacy, Russell and Burch's three principles fall short of providing a comprehensive account of the ethics of animal research. A full account of the ethics of brain organoid research will need to draw on a more comprehensive set of moral principles. One such set of principles has recently been developed by Tom L. Beauchamp and David DeGrazia.^24^ Beauchamp and DeGrazia present six ethical principles of animal research, each of which is intended to be morally uncontroversial. Four of these principles are particularly useful for closing the gaps left by the Three R's. These are the Principle of Sufficient Value to Justify Harm (according to which the anticipated benefits of a research study must be sufficiently weighty to justify the harms to animal research subjects), the Principle of No Unnecessary Harm (according to which researchers should minimize harms to animal subjects), the Principle of Basic Needs (according to which researchers should meet animals' basic needs unless failure to do so is necessary for and morally justified by scientific purposes), and the Principle of Upper Limits to Harm (according to which animal subjects must not be made to endure severe long-term suffering except in rare cases of critically important research.)

These principles suggest three additional limits to organoid research. First, research with conscious (or potentially conscious) brain organoids should proceed only if the anticipated benefits of this research are significant enough to outweigh the expected harms. The greater the expected harm to brain organoids, the greater the expected benefits must be to justify the research. The reverse is also true: the more important the research, the greater the potential harms must be to justify preventing such research from going ahead.

Second, researchers should actively minimize the harms they might inflict through their research. This might involve not only refining experimental procedures to reduce harm, but also conducting research on whatever kind of brain organoids are likely to experience the least suffering. If (as seems plausible) a being's capacity for suffering is tied to the richness and complexity of its mental life, then we can limit the harm we inflict by using brain organoids with the lowest potential degree of consciousness compatible with achieving the goals of the research. In principle, it might be possible to further mitigate harms through gene editing. If brain organoids would develop conscious capabilities in excess of what is required for the research, gene editing could potentially be used to reduce their capacity for consciousness and/or to prevent brain organoids from experiencing pain or other aversive mental states.

Third, in line with the Principle of Upper Limits to Harm, conscious brain organoids should not be made to experience severe long-term suffering unless this is necessary to achieve some critically important scientific goal. As suggested above, it might be possible to mitigate severe suffering by conducting harmful forms of experimentation on brain organoids that possess the lowest possible degree of consciousness and/or have been genetically modified to ease aspects of consciousness that would otherwise contribute to severe suffering.

To summarize, we ought to introduce some limits to research with brain organoids that are or could plausibly be conscious. Such research should proceed only if (a) the research serves a sufficiently important purpose to outweigh the expected costs, including harms to the organoids themselves, (b) the research cannot be conducted using non-conscious organoids or other non-sentient material, (c) researchers use the minimum number of organoids than is required to answer the research question, (d) the organoids used do not have a higher potential capacity for suffering than is necessary to achieve the scientific objectives of the research, (e) the research is designed to minimize possible suffering, and (f) the research would not inflict severe long-term suffering, unless necessary to achieve some critically important purpose.

## Research Limits Beyond Consciousness

So far, we have been assuming that brain organoids are unlikely to develop sophisticated forms of cognition. This assumption seems reasonable, at least insofar as these brain organoids would be unable to interact with their environment; the development of sophisticated brain networks is often thought to require both input (such as sensory input) and output (such as the ability to interact with surroundings). However, researchers are already exploring techniques that could eventually push these boundaries — for example, by wiring brain organoids to muscle tissue,^25^ by connecting brain organoids to controllable robotic “bodies,”^26^ or by implanting human brain organoids into non-human animals' brains.^27^ Researchers have already created “photosensitive” brain organoids, which feature rudimentary eyes and display neural activity when light is shined on them.^28^ It is not implausible to think that sufficiently mature organoids could develop advanced cognitive capacities through interacting with the outside environment.

This possibility has two important implications. The first is that organoids with advanced cognitive capacities might have a wider range of morally relevant interests than organoids that are merely conscious. Cognitively sophisticated beings can develop a wide range of social, emotional, and cognitive needs, and they can suffer these needs go unmet. For example, humans and many non-human animals will suffer if they lack opportunities to socialize with conspecifics, or if they are treated in ways that cause lingering anxiety or fear. Self-consciousness (i.e., the capacity to think of oneself as oneself) has some especially weighty moral implications. Because self-conscious beings can have desires and ambitions for the future, they can be harmed by death even if they are killed painlessly.^29^ To kill a self-conscious being therefore requires a weightier justification than to kill a being that lacks self-consciousness or possesses it only to a rudimentary degree. Self-consciousness might also have greater moral significance; indeed, some argue that it is never appropriate to use self-conscious beings for invasive research.^30^ At a minimum, if brain organ-oids develop advanced cognitive capacities we ought to account for their full range of their welfare needs, not merely narrow interests in avoiding pain or other aversive sensations.

It might be difficult to predict the cognitive functioning or welfare requirements of such organoids. Consistent with existing ethical standards for part-human chimera research,^31^ we should screen human brain organoids for advanced cognitive capacities they could plausibly develop. If brain organoids *do* develop advanced cognitive capacities, researchers should take any associated welfare needs into account. This might involve providing environmental enrichment, and potentially even social opportunities, appropriate to the kinds of beings these brain organoids are.

There is another reason why it matters, morally, if brain organoids develop advanced cognitive capacities: because some cognitive capacities might affect brain organoids' moral status. Moral status is often (and we think plausibly) considered to be a *matter of degree.* There are two versions of this view.^32^ On the first view, we might hold that the interests of all conscious beings deserve equal consideration and yet *also* hold that because different kinds of beings experience different forms of suffering to different degrees, some beings deserve greater protections than others. In line with this view, we might think that cognitively sophisticated brain organoids are susceptible to greater suffering than brain organoids that are merely conscious, and therefore deserve greater protection. On the second view, we might think that the degree to which a being possesses certain cognitive capacities — such as autonomy, sociality, and/or self-consciousness — should directly affect the degree of weight we attach to that being's interests. In line with this latter view, we might think that we ought to attach greater weight to the interests of cognitively sophisticated brain organoids than those that are merely conscious. On either view, research with cognitively sophisticated brain organoids requires a more powerful justification than research with those that lack advanced cognitive abilities.

We have argued that advanced cognitive capacities could give rise to new welfare requirements and potentially increase brain organoids' degree of moral status. Research with cognitively advanced brain organoids should therefore face a further set of research limits. Specifically, research with advanced brain organ-oids should proceed only if (a) they are screened for cognitive capacities they could plausibly develop, (b) any associated welfare requirements are taken into account, (c) brain organoids' cognitive capacities are not more sophisticated than is necessary to achieve the goals of the research, and (d) the research serves a sufficiently important purpose to outweigh the harms to the organoids themselves, taking into account these organoids' (potentially enhanced) degree of moral status.

In applying this framework, we should err on the side of generosity when resolving uncertainty regarding brain organoids' cognitive capacities and/or moral status. All else being equal, it would be worse to mis-takenly treat brain organoids as if they have advanced cognitive capacities (when in fact they lack them) than to mistakenly treat them as if they lack advanced cognitive capacities (and therefore fail to recognise important welfare requirements and/or underestimate their degree of moral status).

## Conclusion

Taken together, our suggestions comprise a moral framework for brain organoid research (Table 1). This framework has three tiers. First, brain organoids that could not plausibly possess consciousness should not face limits beyond those that apply to research with human biological material in general. Second, brain organoids that could possess consciousness should be extended some protections against suffering. Third, advanced brain organoids capable of interacting with the environment should be screened for unexpected cognitive capacities and, all else being equal, have any associated welfare needs respected.

**Table 1:** Proposed research limits

Equivalent stage of human *in vivo* brain development	Research restrictions
Non-conscious brain organoids (e.g., equivalent to fewer than 20 weeks' *in vivo* brain development)	Research should be regulated according to existing frameworks for stem cell and human biospecimen research
Conscious or potentially conscious brain organoids (e.g., equivalent to 20 weeks' *in vivo* brain development or more)	In addition to the above constraints, research should be subject to the following restrictions: The expected benefits of the research must be sufficiently great to justify the moral costs, including potential harms to brain organoids. Conscious brain organoids should be used only if the goals of the research cannot be met using non-sentient material. The minimum possible number of brain organoids should be used, compatible with achieving the goals of the research. Conscious brain organoids should not have greater potential for suffering than is necessary to achieve the goals of the research. Conscious brain organoids must not experience greater harm than is necessary to achieve the goals of the research. Brain organoids should not be made to experience severe long-term harm unless necessary to achieve some critically important purpose.
Brain organoids with the potential to develop advanced cognitive capacities (e.g., mature brain organoids capable of interacting with outside environment.)	In addition to the above constraints, research should be subject to the following restrictions: Brain organoids should be screened for advanced cognitive capacities they could plausibly develop. In general, such assessments should err on the side of overestimating rather than under-estimating cognitive capacities. Cognitive capacities should not be more sophisticated than is necessary to achieve the goals of the research. Welfare needs associated with advanced cognitive capacities should be met unless failure to do so is necessary to achieve the goals of the research. The expected benefits of the research must be sufficiently great to justify the expected or potential harms. This calculation should take into account the implications of advanced cognitive abilities for brain organoids' welfare and moral status.

It is worth emphasising that our proposed research limits are not intended to hamstring the kinds of research that are already underway. There seems to be little risk that the brain organoids already being created are conscious, and we do not necessarily expect consciousness to be achieved until the science has advanced much further than it is today. The prospect of creating organoids with self-consciousness or other advanced cognitive abilities lies still further in the future. Even once these milestones have been reached, our framework is not intended to rule out research with conscious brain organoids; it is merely designed to ensure their welfare is taken into account.

The prospect that brain organoids could one day develop consciousness is ethically concerning. However, the prospect of halting brain organoid research altogether is no less alarming. We have powerful moral reasons to support research that could promote scientific knowledge and increase human well-being. What is needed — and what we have attempted to provide — is a framework that can prevent unethical forms of experimentation without unduly interfering with the field's ability to yield valuable scientific insights.

## References

[1] de Souza N., Editorial, “Method of the Year 2017: Organoids,” Nature Methods 15, no. 1 (2018): 23.

[2] Qian X. et al, “Using Brain Organoids to Understand Zika Virus-Induced Microcephaly,” Development 144, no. 6 (2017): 952-957. 28292840 10.1242/dev.140707 PMC5358105

[3] Mariani J. et al, “FOXG1-Dependent Dysregulation of GABA/Glutamate Neuron Differentiation in Autism Spectrum Disorders,” Cell 162, no. 2 (2015): 375-390. 26186191 10.1016/j.cell.2015.06.034 PMC4519016

[4] Stachowiak E. et al, “Cerebral Organoids Reveal Early Cortical Maldevelopment in Schizophrenia — Computational Anatomy and Genomics, Role of FGFR1,” Translational Psychiatry 7, no. 11 (2017), *available at* < 10.1038/s41398-017-0054-x> (last visited November 15, 2019). PMC5802550 30446636

[5] Bredenoord A.L., Clevers H., and Knoblich J.A., “Human Tissues in a Dish: The Research and Ethical Implications of Organoid Technology,” Science 355, no. 6322 (2017): aaf9414; 10.1126/science.aaf9414 28104841

[6] Trujillo C.A. et al, “Nested Oscillatory Dynamics in Cortical Organoids Model Early Human Brain Network Development,” bioRxiv (2018), *available at* < 10.1101/358622> (last visited November 15, 2019). PMC6778040 31474560

[7] Koplin J. and Savulescu J., “Fresh Urgency in Mapping Out Ethics of Brain Organoid Research,” The Conversation, 11 21, 2018, *available at* <https://theconversation.com/fresh-urgency-in-mapping-out-ethics-of-brain-organoid-research-107186> (last visited November 15, 2019);

[8] Aach J. et al, “Addressing the Ethical Issues Raised by Synthetic Human Entities with Embryo-Like Features,” Elife 6 (2017): e20674; 28494856 10.7554/eLife.20674 PMC5360441

[9] National Health & Medical Research Council, National Statement on Ethical Conduct in Human Research (2007), at section 3.2.

[10] Aach et al., *supra* note 8; Farahany et al., *supra* note 8.

[11] International Society for Stem Cell Research, Guidelines for Stem Cell Science and Clinical Translation (2016), *available at* <http://www.isscr.org/guidelines2016> (last visited November 15, 2019).

[12] Lavazza and Massimini, *supra* note 8.

[13] Rossi G., Manfrin A., and Lutolf M.P., “Progress and Potential in Organoid Research,” Nature Reviews Genetics 19, no. 11 (2018): 671-687. 10.1038/s41576-018-0051-9 30228295

[14] Shepherd J., Consciousness and Moral Status (New York: Routledge, 2018): at 7 Admittedly, this claim is not entirely uncontroversial. For example, it is sometimes argued that moral status extends to entities that have the mere potential to develop into human persons (see e.g. Watt H., “Potential and the Early Embryo,” Journal of Medical Ethics 22, no. 4(1996): 222-226).

[15] Lagercrantz H., “The Emergence of Consciousness: Science and Ethics,” Seminars in Fetal and Neonatal Medicine 19, no. 5 (2014): 300-305. 25160864 10.1016/j.siny.2014.08.003

[16] Lee S.J. et al, “Fetal Pain: A Systematic Multidisciplinary Review of the Evidence,” JAMA 294, no. 8 (2005): 947-954. 16118385 10.1001/jama.294.8.947

[17] Brugger E.C., “The Problem of Fetal Pain and Abortion: Toward an Ethical Consensus for Appropriate Behavior,” Kennedy Institute of Ethics Journal 22, no. 3 (2012): 263-287. 23285794 10.1353/ken.2012.0014

[18] Lavazza and Massimini, *supra* note 8.

[19] Bradshaw R.H., “Consciousness in Non-Human Animals: Adopting the Precautionary Principle,” Journal of Consciousness Studies 5, no. 1 (issue number?) (1998): 108-114;

[20] Tooley M., “Personhood,” in Kuhse H. and Singer P., eds, A Companion to Bioethics (Oxford: Blackwell, 2009): 127-139.

[21] For an overview of such views, see: Andrews K. et al, Chimpanzee Rights: The Philosophers' Brief (New York: Routledge, 2018): at 41-60.

[22] DeGrazia D., “The Moral Status of Animals and their Use in Research: a Philosophical Review,” Kennedy Institute of Ethics Journal 1, no. 1 (1991): 48-70; 11645700 10.1353/ken.0.0112

[23] Russell W.M.S. and Burch R.L., The Principles of Humane Experimental Technique (London: Methuen, 1959).

[24] Beauchamp T. and DeGrazia D., Principles of Animal Research Ethics (Oxford: Oxford University Press, 2019).

[25] Giandomenico S.L. et al, “Cerebral Organoids at the Air–Liquid Interface Generate Diverse Nerve Tracts with Functional Output,” Nature Neuroscience 22, no. 4 (2019): 669-679. 30886407 10.1038/s41593-019-0350-2 PMC6436729

[26] Cohen J., “Neanderthal Brain Organoids Come to Life,” Science 360, no. 6395 (2018): 1284. 29930117 10.1126/science.360.6395.1284

[27] Mansour A.A. et al, “An in vivo Model of Functional and Vascularized Human Brain Organoids,” Nature Biotechnology 36 (2018): 432-441. 10.1038/nbt.4127 PMC6331203 29658944

[28] Quadrato G. et al, “Cell Diversity and Network Dynamics in Photosensitive Human Brain Organoids,” Nature 545, no. 7652 (2017): 48-53. 28445462 10.1038/nature22047 PMC5659341

[29] Singer P., Practical Ethics (Cambridge: Cambridge University Press, 2011), at 112 See also McMahan J., The Ethics of Killing: Problems at the Margins of Life (New York: Oxford University Press, 2002), at 194-203. Note that self-consciousness could be a matter of degree — in which case the wrongness of killing a self-conscious being might scale according to the degree of self-consciousness. See e.g. DeGrazia D., “Self-awareness in Animals,” in Lurz R. ed, The Philosophy of Animal Minds (Cambridge: Cambridge University Press, 2009): 201-217.

[30] See e.g. Hyun I., Bioethics and the Future of Stem Cell Research (New York, NY: Cambridge University Press, 2013), chapter 6.

[31] Hyun I. et al, “Ethical Standards for Human-to-Animal Chimera Experiments in Stem Cell Research,” Cell Stem Cell 1 (2007): 159-163; 18383627 10.1016/j.stem.2007.07.015

[32] DeGrazia D., “Moral Status as a Matter of Degree?” The Southern Journal of Philosophy 46, no. 2 (2008): 181-198.

